# Worsening Mental Health and Self-Rated Health in Older Incarcerated Persons During the COVID-19 Pandemic

**DOI:** 10.1093/geroni/igab046.1861

**Published:** 2021-12-17

**Authors:** Lisa Barry, Deborah Noujaim, Alexandra DePalma, Emil Coman, Dorothy Wakefield

**Affiliations:** 1 University of Connecticut Center on Aging, Farmington, Connecticut, United States; 2 UConn Health, Farmington, Connecticut, United States

## Abstract

Incarcerated persons age 50 and older comprise one of society’s most vulnerable groups given high rates of chronic illness, estrangement from family/friends, and suicide. Consequently, the mental health impact of COVID-19 on this population may be especially salient. Using data from the ongoing Aging Inmates’ Suicidal Ideation and Depression study (Aging INSIDE), we determined change in older incarcerated persons’ mental health (anxiety and depression symptoms) and change in self-rated health (SRH) from before to during the COVID-19 pandemic, and evaluated how these variables were related. Of the 202 still-incarcerated Aging INSIDE participants, 157 (77%) completed Check-In Surveys between August-September 2020. Participants were 96% male, racially diverse (41% White, 41% Black, 18% Hispanic/Other) and average age was 56.0(±5.8) years. From before to during the COVID-19 pandemic, average anxiety symptom scores, assessed by the GAD-7, increased (worsened) (from 6.4±5.7 to 7.8±6.6; p<0.001), average depressive symptoms scores, measured by the PHQ-8, increased (worsened) (from 5.5±6.0 to 8.1±6.5; p<0.001), and average SRH decreased (worsened) (from 3.0±0.2 to 2.6±0.2; p<0.001). Worsening anxiety led to worsening depressive symptoms (direct effect = 0.339; p<0.05). A mediation model controlling for age, race, chronic conditions, years until release, and change in social support score found a total effect of change in anxiety on SRH change of -0.04 (p<0.001), of which 34.2% flows indirectly through change in depression (p<0.001). Older incarcerated persons experienced worsening mental health and SRH during the COVID-19 pandemic. Future research will determine if mental health and SRH improve following vaccination and return to “normal” procedures.

